# Distribution and Function of Glycosaminoglycans and Proteoglycans in the Development, Homeostasis and Pathology of the Ocular Surface

**DOI:** 10.3389/fcell.2020.00731

**Published:** 2020-08-07

**Authors:** Sudan Puri, Yvette M. Coulson-Thomas, Tarsis F. Gesteira, Vivien J. Coulson-Thomas

**Affiliations:** ^1^College of Optometry, University of Houston, Houston, TX, United States; ^2^Molecular Biology Section, Department of Biochemistry, Universidade Federal de São Paulo, São Paulo, Brazil; ^3^Optimvia, LLC, Batavia, OH, United States

**Keywords:** cornea, wound healing, lumican, keratan sulfate, decorin

## Abstract

The ocular surface, which forms the interface between the eye and the external environment, includes the cornea, corneoscleral limbus, the conjunctiva and the accessory glands that produce the tear film. Glycosaminoglycans (GAGs) and proteoglycans (PGs) have been shown to play important roles in the development, hemostasis and pathology of the ocular surface. Herein we review the current literature related to the distribution and function of GAGs and PGs within the ocular surface, with focus on the cornea. The unique organization of ECM components within the cornea is essential for the maintenance of corneal transparency and function. Many studies have described the importance of GAGs within the epithelial and stromal compartment, while very few studies have analyzed the ECM of the endothelial layer. Importantly, GAGs have been shown to be essential for maintaining corneal homeostasis, epithelial cell differentiation and wound healing, and, more recently, a role has been suggested for the ECM in regulating limbal stem cells, corneal innervation, corneal inflammation, corneal angiogenesis and lymphangiogenesis. Reports have also associated genetic defects of the ECM to corneal pathologies. Thus, we also highlight the role of different GAGs and PGs in ocular surface homeostasis, as well as in pathology.

## Introduction

**FIGURE 1:**
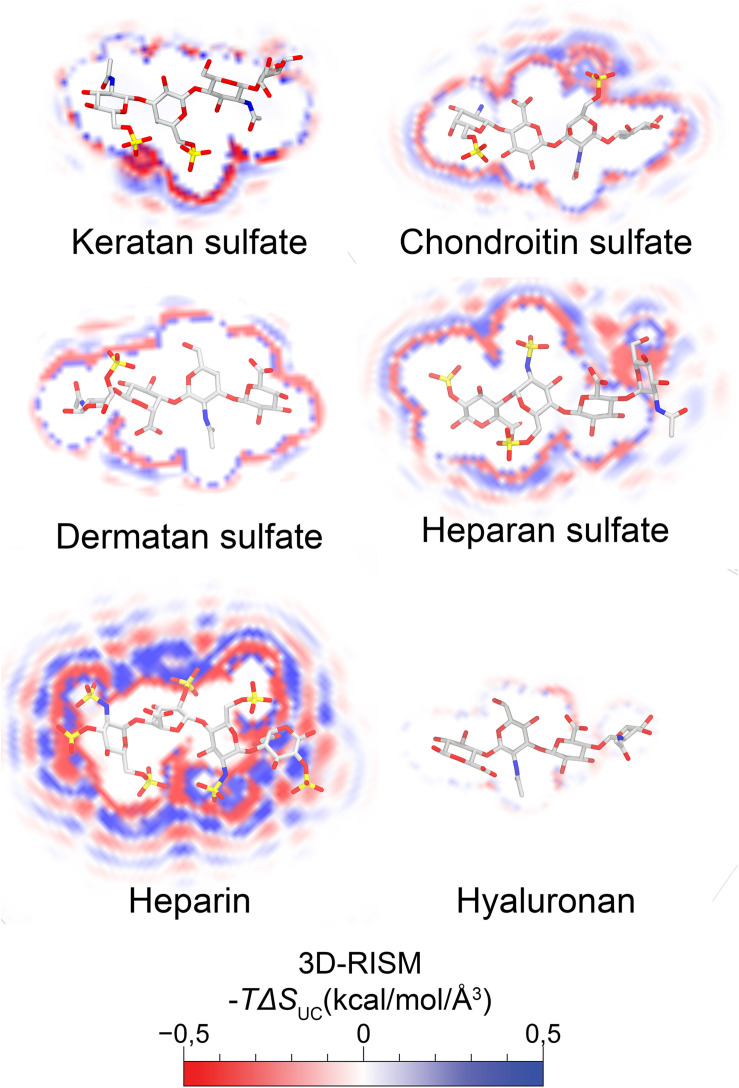
Molecular reconstruction of thermodynamic maps of water entities around a representative tetrasaccharide of the different GAGs. Molecular reconstruction of thermodynamic maps of water entities were depicted additively by combining oxygen and hydrogen against a solute. Solute-solvent interaction description was calculated by 3D-RISM (three-dimensional reference interaction site model with Universal Correlation, UC) (Luchko et al., 2010; Nguyen et al., 2019). The displacement of water molecules favorably modulates the free energy of protein-ligand complex binding. A cross section at the center of mass is represented in the *x*-axis for each tetrasaccharide with a licorice representation of each GAG in an overlay. The thermodynamic maps of water entities can be directly correlated with the electrostatic charges of each tetrasaccharide unit and the specificity with which they bind to their binding partners.

The ECM components are not only space fillers, as believed for many decades, but are actually physiologically active components that play an important role in development, homeostasis and pathology (Badylak et al., 2009; Frantz et al., 2010; Clause and Barker, 2013). PGs and GAGs are ubiquitous components of the ECM and are particularly enriched in connective tissues (Varki et al., 1999). PGs are composed of a core protein covalently linked to at least one GAG side chain and can be classified into different groups based on their core protein and/or the composition of GAG side chains (Hardingham and Fosang, 1992; Iozzo, 1998). GAGs are unbranched polysaccharides with repeating disaccharide units composed of a hexosamine (D-GlcNAc or D-GalNAc) and a hexuronic acid (D-GlcA, L-IdoA, or D-Gal in the case of KS) (Anseth, 1969; Varki et al., 1999; Mikami and Kitagawa, 2017). Based on the composition of the repeating disaccharide unit, GAGs are subdivided into CS, DS, KS, HS/heparin (HEP) and HA (a stick representation of each GAG is shown in Figure 1) (Anseth, 1969; Varki et al., 1999). During biosynthesis, GAG chains undergo secondary modifications by the actions of sulfotransferase and epimerase enzymes (Mikami and Kitagawa, 2017). The spatiotemporally controlled expression of these enzymes enables cells to fine-tune sugar heterogeneity (Varki et al., 1999). Sulfotransferases use the universal sulfate donor PAPS to add 2-, 6- and 3-*O* sulfates to HS/HEP and 2-, 4-, and 6-*O* sulfates to CS/DS, while the epimerases convert GlcA to IdoA (Mencio et al., 2015). Thus, GAG biosynthesis is not template driven, which means there is significant variation in sulfation patterns and glucuronic/IdoA content among the synthesized GAGs, which are essential in determining their physical properties (Varki et al., 1999; Ohtsubo and Marth, 2006; Afratis et al., 2013). The presence of carboxylate and sulfate groups provides GAGs with highly negative charges and means they attract and retain water, which also directly correlates with their physiological properties (Rozario and DeSimone, 2010). These water molecules play an essential role in mediating interactions between GAG and protein binding sites (Warshel et al., 1986; Sarkar et al., 2016; Cui et al., 2018). In Figure 1 we demonstrate the calculated water energy density distributions for the different GAGs, specifically the force with which each GAG interacts with water molecules. The representative tetrasaccharide unit of heparin has the highest solvation energy, while the tetrasaccharide unit in HA has the lowest. Recent studies have shown that GAG chains preferentially bind to ligands that will lower their desolvation energy and water molecules participate in many GAG-protein interactions within the binding site (Sarkar et al., 2016; Cui et al., 2018). Figure 1 shows the solvent-solute energies of a representative tetrasaccharide for each GAG, calculated using the three-dimensional reference interaction site model (3D-RISM) theory. These energy maps function as a predictive of the specificity of the interaction of each GAG with their binding partners (Sugita et al., 2020).

Glycosaminoglycans and PGs have been shown to participate and/or regulate various signaling pathways, such as, the TGF-β, JNK/p38, FAK, and ERK pathways. TGF-β is a family of dimeric proteins with three isoforms – TGF-β1, TGF-β2, and TGF-β3 expressed by corneal and limbal epithelia, conjunctiva and stromal keratocytes (Saika et al., 2004; Chen et al., 2006). TGF-β receptor-III (betaglycan) is the most abundant membrane PG receptor containing HS and CS chains (Cheifetz et al., 1988; Chen et al., 2006). The HS regulates the binding of betaglycan to TGF-β and the deficiency or enzymatic removal of HS in epithelial cells reduces the degradation of TGF-β1 (Chen et al., 2006). TGF-β plays an important role in ocular surface homeostasis and pathology as it induces the synthesis and secretion of ECM components, promotes adhesion complex formation with corneal epithelial cells, increases protease inhibitor expression and recruits monocytes/neutrophils during inflammation (Gassner et al., 1997; Ljubimov and Saghizadeh, 2015). The TGF-β-receptor complex activates the Smad signaling pathway and other non-Smad pathways involving ras/MEK/ERK, RhoA, stress kinases (JNK and p38MAPK), and PI3K/AKT (Bhowmick et al., 2001; Santander and Brandan, 2006). The ERK signaling pathway requires the HSPG syndecan-4 for Cellular Communication Network Factor 2 and type I collagen expression (Leask, 2010; Nourian et al., 2019). TGF-β also induces the differentiation of fibroblasts to myofibroblasts via the FAK-MEKK1-JNK pathway (Liu et al., 2007). TGF-β activates FAK, which induces JNK-dependent actin stress fiber formation and expression of profibrotic genes in fibroblasts leading to ECM remodeling (Liu et al., 2007; Leask, 2010).

The ocular surface, including the cornea, corneoscleral limbus, conjunctiva, tear producing glands and tear film, form the interface between the external and internal environment of the eye. The unique organization of ECM components throughout the ocular surface is essential for maintaining healthy tissue, and therefore for enabling vision. The ocular surface is protected from external chemicals and microbes by mucin glycoproteins rich in *O*-glycans, which are synthesized by corneal and conjunctival epithelial cells (Brockhausen et al., 2018). Since the initial study of GAGs in corneal disorders in 1969 (Anseth, 1969), a plethora of studies have investigated the role of GAGs and PGs in the ocular surface of various species including humans. For example, HSPGs are constituents of cell-cell and cell-basement membrane junctions that are important for the barrier function of the corneal epithelium and endothelium, while small leucine-rich PGs (SLRPs) are necessary for the organization of collagen fibers within the stroma and are therefore necessary for maintaining corneal transparency (Varki et al., 1999). More recently, important roles have been suggested for the ECM in regulating limbal stem cells, corneal innervation, corneal inflammation, angiogenesis and lymphangiogenesis (Cursiefen et al., 2004; Gesteira et al., 2017; Pal-Ghosh et al., 2017; Sun et al., 2019). Moreover, ECM components have also been shown to be important constituents of the tear film (Baier et al., 1990; De Souza et al., 2006). Reports have also associated genetic defects to abnormalities in the ECM leading to corneal opacity and blindness (Clause and Barker, 2013). Herein, we will outline the roles of GAGs and PGs in the different compartments of the ocular surface and how they correlate with pathology.

## Keratan Sulfate Proteoglycans (KSPGs)

Keratan sulfate is composed of a repeating disaccharide unit made up of GlcNAc and a Gal, more specifically 3Galβ1-4GlcNAcβ1, and can be sulfated at carbon position 6 (C6) of the Gal or GlcNAc monosaccharides. Although all GAGs are bound to PGs via the tetrasaccharide linkage region Xyl-Gal-Gal-GlcNAc, KS is an exception and has a unique linkage region which gives rise to different types of KS, namely KS types I, II, and III (Funderburgh, 2000). More specifically, KS type I attaches to an Asn residue on the core protein via a complex N-linked branched oligosaccharide. KS type II attaches to a serine or threonine (Ser/Thr) amino acid on the core protein via an O-linked glycan (α-Gal-Ser/Thr). KS type III attaches to a Ser amino acid on the core protein via a mannose-Ser linkage (Funderburgh, 2000). The cornea is the tissue with the highest KS content and is the richest source of type I KSPGs, which were originally identified in the cornea in 1939 (Suzuki, 1939; Funderburgh, 2000; Quantock et al., 2010). Normal human corneas contain 15 μg of KS per mg dry weight with ∼14 disaccharides per chain consisting of ∼4% unsulfated, 42% monosulfated, and 54% disulfated disaccharides (Plaas et al., 2001). The enzymatic sulfation of KS by GlcNAc-6-*O*-sulfotransferase (GlcNAc6ST) is required for normal collagen matrix biosynthesis and organization in the cornea (Hayashida et al., 2006). Reduced activity of GlcNAc6ST, which catalyzes the transfer of sulfate to position 6 of terminal GlcNAc, results in low-sulfated KS synthesis and accumulation in macular corneal dystrophy (Hasegawa et al., 2000). In normal human corneas, KS is predominantly found in the central cornea and gradually decreases toward the limbus–sclera regions (Borcherding et al., 1975). Loss of KS in corneas of β-1,3-NAcetylglucosaminyltransferase-7 *null* mice leads to corneal thinning (Littlechild et al., 2018). Interestingly, KS deficient mice significantly up-regulate CS throughout the corneal stroma through a compensatory mechanism (Littlechild et al., 2018).

**FIGURE 2:**
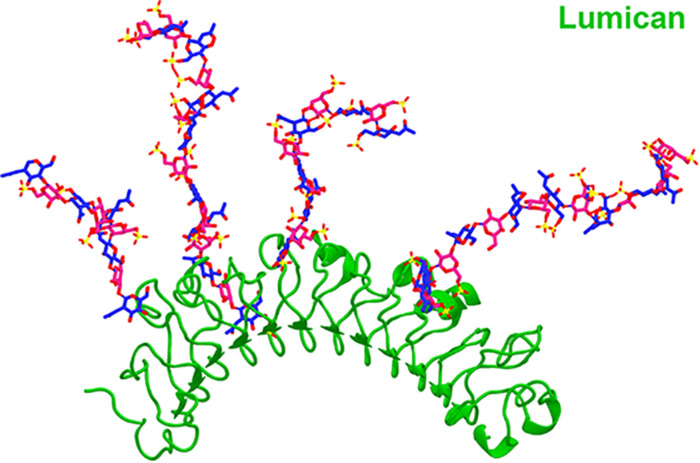
A cartoon representation of the SLRP lumican containing four KS side chains. The structure of lumican was modeled on the crystalized structure of decorin and presented as a cartoon representation of human lumican (green). The KS side chains at the *N*-glycosilation sites at N88, N127, N160 and N252 are represented as a sticks (*N*-acetyl glucosamine in pink, galactose in blue, oxygen in red and sulfate in yellow).

**FIGURE 3:**
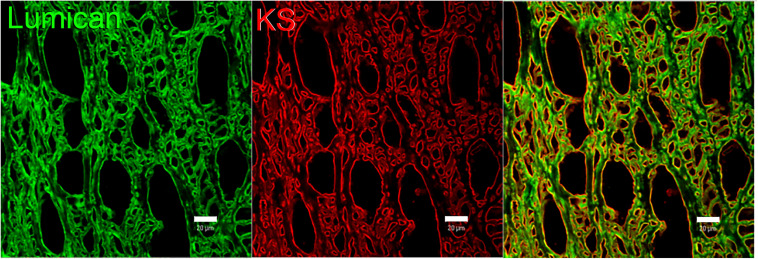
Representative image of lumican and KS imunolocalized in human corneas. Lumican (green) and KS (red) were immunolocalized in a sagittal section of a normal human cornea. Lumican was detected with a monoclonal anti-lumican antibody, clone 1F12B10, produced by our group, and KS was detected using anti-KS clone 5D4, kindly provided by Prof. Bruce Caterson. Scale bar represents 20 μm.

**FIGURE 4:**
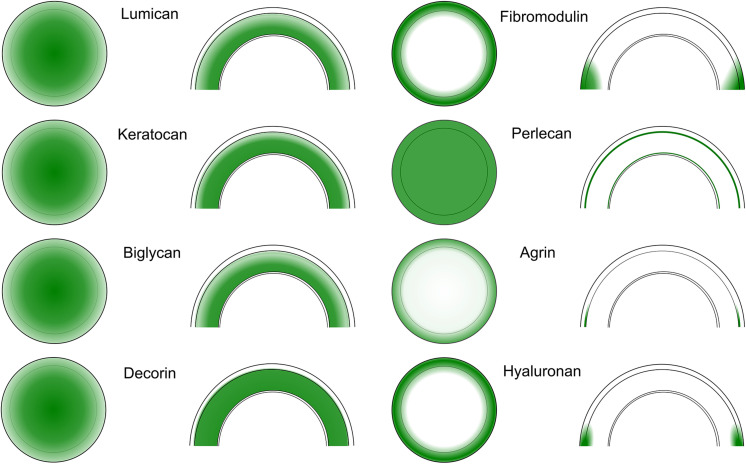
Spatial distribution of SLRPs, HSPGs and HA in the cornea and limbal region. Spatial distribution maps showing the distribution of the SLRPs lumican, keratocan, decorin, biglycan, and fibromodulin, the HSPGs perlecan and agrin, and HA, all represented in green, in the cornea and limbal region. A frontal view of the whole cornea and limbal region is shown on the left of each panel, with the inner circle demarcating the cornea/limbus interface. The corresponding image on the right show a cross-sectional view of the cornea with the limbal regions toward the bottom. The lines demarcate the different layers of the cornea, specifically the upper line represents the intersection between the epithelium and the stroma, with the other line representing the intersection between the stroma and the endothelium. The intensity of the green color correlates with expression levels, with the darker color representing higher expression.

The KS in corneas is mostly bound to SLRPs such as lumican, keratocan, fibromodulin, and osteoglycin/mimecan, which have been shown to regulate corneal development, corneal transparency and wound healing. Lumican belongs to the SLRP family and has four N-linked glycosylation sites which can contain KS side chains in humans (Chakravarti et al., 1995). A representation of lumican containing four KS side chains is shown in Figure 2. The characteristic leucine-rich repeats in the protein core mediate binding to other extracellular components like collagen (Danielson et al., 1997; Chakravarti et al., 1998; Smith and Hassell, 2006). The structure of lumican is homologous to decorin; a superhelix with a horseshoe or arch-like structure having β-sheets at the concave surface and α-helices at the convex surface (Chakravarti et al., 1995; Weber et al., 1996; Iozzo, 1997). Our group has modeled the structure of lumican using decorin as a template and the location of KS attachment sites can be seen on the convex side of the molecule (Figure 2). This shape allows for accommodation of a collagen fibril within the concave surface of lumican (and other SLRPs), and the KS side chains that are bound to the convex side project outwards. These KS side chains, which due to their high anionic charge retain water, are important for the normal assembly and growth of collagen fibrils (Iozzo, 1997; Chakravarti et al., 1998). Immunostaining of human corneas with anti-lumican (green) and anti-KS (red) antibodies demonstrates how the KS side chains project outwards into the “empty” space (Figure 3). Specifically, a thin line of KS (represented in red) can be seen lining the outer surfaces of the stromal lamellae (Figure 3). In adult mouse corneas, immunohistochemistry shows an increasing concentration of lumican from low levels of non-specific staining in the epithelium to markedly increased levels in the posterior third of the stroma (Figure 4) (Chakravarti et al., 2000; Bouhenni et al., 2013). Studies in chick corneal development have shown that the switch from the glycoprotein form of lumican (containing polylactosamine – non-sulfated KS) side chains, at embryonic day 7 (E7) to the PG form of lumican (with sulfated KS side chains) after E15 is well correlated with development of corneal transparency (Cornuet et al., 1994; Dunlevy et al., 2000). Similarly, the switch to lumican containing KS side chains occurs concomitantly with corneal transparency and eye opening in mice (Ying et al., 1997). The important role lumican plays in maintaining corneal transparency came to light with the generation of lumican *null* mice (Chakravarti et al., 1998; Saika et al., 2004). These studies revealed that lumican is necessary for the correct alignment of collagen fibers and for maintaining interfibrillar collagen spacing (Chakravarti et al., 1998, 2000). Lumican *null* mice have collagen fibers with a larger fibril diameter, abnormal fibril packing, and irregular lamellar organization particularly in the posterior stroma (Chakravarti et al., 1998, 2000).

**FIGURE 5:**
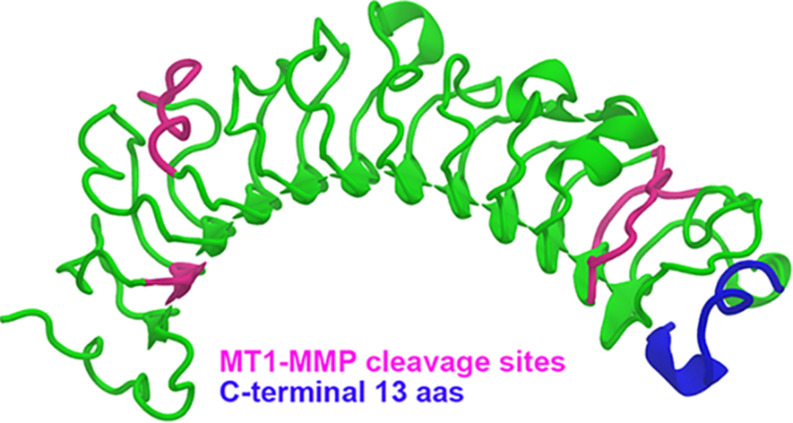
A cartoon representation of the lumican core protein with indicated MT1-MMP cleavage sites. A cartoon representation of human lumican (green) modeled using the structure of decorin (pdbID 1XDC) as a scaffold. MT1-MMP cleavage sites are depicted in pink in order to represent the lumican products that would be produced upon MT1-MMP cleavage. The c-terminal 13 aas of lumican, which have been shown to promote corneal wound healing, have been shown in blue.

The role of lumican in corneal wound healing was evidenced by delayed closure of epithelial defects in lumican-*null* mice *in vivo* and slower scratch closure after addition of an anti-lumican antibody *in vitro* (Saika et al., 2000). Lumican expression during corneal wound healing contributes to important processes like epithelial cell migration and adhesion (Saika et al., 2000), neutrophil and macrophage recruitment (Funderburgh et al., 1997; Vij et al., 2004), proliferation and apoptosis of stromal keratocytes (Vij et al., 2004). Lumican is expressed by stromal keratocytes and not by the corneal epithelium in normal corneas; however, it is expressed transiently and ectopically by mouse corneal epithelial cells during the process of epithelial wound healing (Saika et al., 2000). Studies have suggested that the functions of lumican may be mediated by the interaction of lumican with Fas/Fas ligand (Vij et al., 2005), TLR4 (Lohr et al., 2012) or Transforming growth factor-β receptor 1 (ALK5) (Yamanaka et al., 2013). Recently, the cleavage of lumican, for example by matrix metalloproteinase 14 (MMP14), has been shown to generate physiologically active products that have been named lumikines by the Kao group (Yamanaka et al., 2013; Gesteira et al., 2017). Thus, upon wound healing, lumican throughout the cornea could serve as a storage pool of signaling molecules that are mobilized with the release of certain MMPs. A representation of MT1-MMP cleavage sites on the lumican core protein can be seen in Figure 4. Interestingly, a C-terminal product of lumican, which can be generated for example by MT1-MMP cleavage (Figure 5), has been shown to bind to ALK5 and promote corneal epithelial wound healing, increasing both epithelial cell migration and proliferation (Yamanaka et al., 2013; Gesteira et al., 2017). Although lumican expression is important during the process of wound healing, persistent up-regulation of lumican beyond wound closure can lead to reduced adhesion of corneal epithelial basal cells, as seen in streptozotocin-induced diabetic rats (Yamamoto et al., 2018).

**FIGURE 6:**
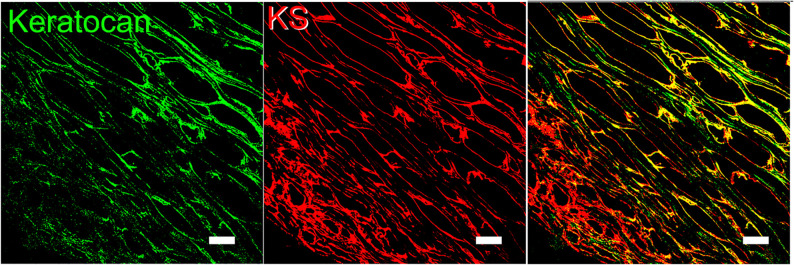
Representative image of keratocan and KS imunolocalized in human corneas. Immunolocalization of keratocan (green) and KS (red) in a sagittal section of normal human cornea. Keratocan was detected with a monoclonal anti-keratocan antibody and KS was detected using anti-KS kindly provided by Prof. Bruce Caterson. Scale bar represents 20 μm.

Keratocan is another member of the SLRP family found in the corneal stroma, and has a protein sequence that is similar to lumican, containing putative KS side chains (Dunlevy et al., 1998). A representative image of keratocan (green) and KS (red) staining in the human cornea can be seen in Figure 6. In the cornea, keratocan is solely expressed by keratocytes, thus it is commonly used as a phenotypic marker for stromal keratocytes (Figure 4) (Kao and Liu, 2002). Because the expression of keratocan is restricted to stromal keratocytes, Keratocan-Cre (Kera-Cre) transgenic driver mice (not inducible) and, recently, the doxycycline inducible Keratocan-rtTA (KeraRT) driver mouse line have been generated to ablate genes specifically in stromal keratocytes (Kao, 2006; Zhang et al., 2017). Lumican has been shown to regulate the expression of keratocan. Specifically, lumican-*null* mice (*in vivo*) and lumican knockdown using siRNA in cells (*in vitro*) both present reduced keratocan expression (Carlson et al., 2005). Furthermore, keratocan transcriptional activity is increased after the expression of lumican is rescued in lumican-*null* mice (Carlson et al., 2005), and keratocan expression is increased at both protein and mRNA levels when lumican is overexpressed in wild-type mouse corneas (Carlson et al., 2005). During corneal wound healing, if keratocytes are activated into a fibroblast or myofibroblast phenotype by FGF-2/heparin sulfate (HS) or TGF-β1, the synthesis of keratocan and lumican is downregulated via activation of the Jun N-terminal kinase-signaling pathway (Chen et al., 2011). KS biosynthesis, chain length and sulfation are significantly reduced in these activated fibroblasts and myofibroblasts when compared to naïve keratocytes (Funderburgh et al., 2003).

Studies in a mouse model of LPS-induced keratitis have shown that lumican and keratocan can regulate the innate immune response. Lumican and keratocan double knockout mice receiving intrastromal LPS present a less intense increase in corneal thickness and cloudiness, and reduced stromal neutrophil invasion when compared to wild-type mice (Carlson et al., 2005). In contrast to lumican-*null* mice, keratocan-*null* mice have thin but clear corneal stromas with abnormal collagen fibril spacing (Carlson et al., 2005). Interestingly, mutations in the keratocan gene leading to a single amino acid substitution or a C-terminal truncation cause flattening of the anterior corneal curvature in humans, a condition called cornea plana (Pellegata et al., 2000).

Fibromodulin, another SLRP with putative KS side chains, is also involved in regulating collagen fibrillogenesis in the peripheral cornea. It is expressed in the central cornea and limbus during development but becomes restricted to the limbus and sclera in adult mice (Figure 4) (Chen et al., 2010). This specific expression pattern during development is correlated with the change in corneal curvature and change in the axial length of the eye during the normal emmetropization process (Chen et al., 2010). Although fibromodulin-*null* mice have normal eye axial length, fibromodulin and lumican have synergistic effects during the emmetropization process in the region-specific regulation of collagen fibrils as shown in combined lumican/fibromodulin-*null* mice (Chakravarti et al., 2003).

Mimecan, another SLRP with putative KS side chains, exists in the corneal stroma as a KSPG and is mostly found in its non-sulfated form in other connective tissues (Funderburgh et al., 1997). Unlike other SLRPs, mimecan seems to have minimal contribution toward the structure and organization of collagen fibrils in the mouse cornea (Beecher et al., 2005). Studies using mimecan-*null* mice found no significant changes in corneal thickness or clarity, but divergent results were found regarding the role of mimecan in regulating collagen fibril thickness (Tasheva et al., 2002; Beecher et al., 2005). On average, collagen fibrils in corneas of mimecan-*null* mice were thicker when compared to wild-type littermates using transmission electron microscopy ultrastructural analyses (Tasheva et al., 2002). However, a low-angle synchrotron x-ray diffraction study showed that the mean collagen fibril diameter, collagen fibril spacing and collagen fibril organization throughout the cornea of mimecan-*null* mice were comparable to those of wt mice (Beecher et al., 2005).

## Chondroitin Sulfate Proteoglycans (CSPGs)

Chondroitin sulfate is composed of repeating disaccharide units of GlcA and GalNAc with sulfation at various positions. Chondroitin sulfate A (chondroitin-4-sulfate) is sulfated at carbon 4 of the GalNAc sugar, chondroitin sulfate C (chondroitin-6-sulfate) is sulfated at carbon 6 of the GalNAc sugar, chondroitin sulfate D (chondroitin-2,6-sulfate) is sulfated at carbon 2 of the GlcA and 6 of the GalNAc sugar, and chondroitin sulfate E (chondroitin-4,6-sulfate) is sulfated at carbons 4 and 6 of the GalNAc sugar (Mikami and Kitagawa, 2013). In the rat cornea, CS expression is high at birth and decreases with postnatal development (Koga et al., 2005). In normal human corneas there are 8 μg of CS/DS per mg dry weight with ∼40 disaccharides per chain, which decreases to ∼15 disaccharides in corneas affected by macular corneal dystrophies (Plaas et al., 2001). CSPGs are important ECM components with diverse functions in tissues, such as regulating cell adhesion, cell growth, neuronal plasticity, neuronal regeneration and cell migration, and form the bulk of the majority of connective tissues (Coulson-Thomas et al., 2008, 2016; Silver et al., 2013; Silver and Silver, 2014; Smith et al., 2015; Gesteira et al., 2016; Pai et al., 2018; Pudełko et al., 2019).

Lubricin, also known as proteoglycan 4 (PRG4) or superficial zone protein, acts as a boundary lubricant anti-adhesive component in tissues, protecting them against frictional forces (Bao et al., 2011). The lubricin in human synovial fluid consists of glycoprotein isoforms that may or may not have CS and/or KS side chains (Lord et al., 2012). Lubricin is produced by ocular surface epithelia, including the corneal and conjunctival epithelia, and the lacrimal and meibomian glands (Cheriyan et al., 2011; Schmidt et al., 2013). Lubricin reduces friction and protects the cornea and conjunctiva against significant shear forces generated by the eyelid during blinking. Hence, lubricin-*null* mice present a dry eye phenotype with ocular surface damage and positive corneal fluorescein staining due to lack of protective lubrication (Schmidt et al., 2013). A randomized clinical trial of recombinant human lubricin in moderate dry eye treatment produced significant improvement in signs and symptoms when compared to HA (Lambiase et al., 2017). Further studies with lubricin could provide promising management options for dry eye disease.

Aggrecan is a large bottlebrush-shaped PG of the lectican family which forms complexes with HA (Kiani et al., 2002; Ng et al., 2003). Aggrecan has three highly conserved globular domains and three less conserved extended domains in different species (Kiani et al., 2002). Aggrecan contains multiple CS side chains and few KS side chains in all species except in mice where aggrecan has a truncated core protein devoid of a KS-rich region (Watanabe et al., 1995). Because of the highly absorbent and lubricating properties of aggrecan, it acts as a shock absorber in cartilage (Chandran and Horkay, 2012). Aggrecan has been reported to be expressed during development as well as in adult corneas of Wistar rats (Koga et al., 2005). Similarly, in human corneas, aggrecan is immunolocalized mostly in the corneal and conjunctival epithelia, with weak staining in the anterior corneal stroma and moderate staining in the scleral stroma (Bouhenni et al., 2013). Although aggrecan is known to be expressed in the cornea, its function remains to be elucidated. To further understand the role of aggrecan in the ocular surface, cmd mice could be used, which are aggrecan knockout mouse models with a functional null mutation of the aggrecan gene (Watanabe et al., 1994).

Versican is another large CSPG of the lectican protein family, which undergoes alternative splicing and occurs as four isoforms, namely V0, V1, V2, and V3, where V0 is full length versican (Zimmermann and Ruoslahti, 1989; Ito et al., 1995). Versican has an HA-binding region at the N-terminus and several CS attachment regions where V0 has both CS-α and CS-β, V1 has CS-β, V2 has CS-α, and V3 does not have any CS attached (Ito et al., 1995). Versican is a major PG in the dermis and plays an important role in skin wound healing. In particular, the V3 isoform promotes normal dermal fibroblast-myofibroblast transition (Hattori et al., 2011). Studies have suggested that versican is involved in corneal development: in the rat cornea, versican isoforms are highly expressed at birth but larger isoforms (V0) are replaced by smaller isoforms during early postnatal development (P1–P14) and become undetectable in adulthood, while the V1 and V2 isoforms remain unchanged (Koga et al., 2005). Versican has been immunolocalized in the subepithelial region of the anterior limbus in human corneas (Schlötzer-Schrehardt et al., 2007). Although many studies have identified versican in the corneas of multiple species, a function has still to be attributed to it. Therefore, as is the case for aggrecan, further studies are needed to unveil the function of versican in the ocular surface.

## Dermatan Sulfate Proteoglycans (DSPGs)

Dermatan sulfate, previously known as chondroitin sulfate B, has the same basic composition as CS; however, the GlcA is epimerized to IdoA by glucuronyl C5-epimerase (Malmström and Fransson, 1975). Thus, similarly to CS, DS is a copolymer of alternating IdoA-GalNAc(4S) (iA unit), IdoA(2S)-GalNAc(4S) (iB unit), or IdoA-GalNAc(4S,6S) (iE unit) units (Maccarana et al., 2009). DS is predominantly present in the skin and plays an important role in wound healing (Penc et al., 1998). DSPGs present in the corneal stroma are involved in controlling the interfibrillar spacing and lamellar adhesion properties of corneal collagens (Maccarana et al., 2009).

Decorin is a member of the SLRP family with three potential GAG attachment sites, but generally only one DS/CS chain is linked to the serine residues in the N-terminal region (Weber et al., 1996). Interaction of decorin and its side chain with growth factors and other proteins helps in the regulation of various processes, such as collagen fibrillogenesis, angiogenesis, autophagy and wound healing (Gubbiotti et al., 2016). Decorin-deficient mice exhibit skin fragility, tendon weakness and lower lung airway resistance due to abnormal collagen fibrillogenesis (Danielson et al., 1997). Mice lacking decorin DS chains, due to mutation of the serine residue at the site of DS substitution, do not exhibit such anomalies (Moffatt et al., 2017). In the cornea, decorin is abundantly expressed in the stroma (Figure 4) and accumulation of a truncated form of decorin causes stromal opacity in congenital stromal corneal dystrophy (Bredrup et al., 2005, 2010; Rødahl et al., 2006). However, decorin-*null* mice present only a few large and irregular fibrils, therefore it has been proposed that decorin alone does not significantly affect corneal collagen fibril phenotype (Danielson et al., 1997; Zhang et al., 2009). Decorin inhibits TGF-β by binding to its core protein in the latent complex leading to inhibition of myofibroblast differentiation and scar formation (Ständer et al., 1999; Tandon et al., 2010). Decorin has been tested as an anti-fibrotic agent in the cornea significantly reducing corneal opacity in a murine model of *Pseudomonas* keratitis (Hill et al., 2018). The Chinnery group further investigated the use of exogenous decorin to promote corneal nerve regeneration after a corneal epithelial abrasion injury and found that human recombinant decorin promotes corneal nerve regeneration (Wu et al., 2020). It was hypothesized that decorin promotes corneal nerve regeneration via the activation of dendritic cells (Wu et al., 2020).

Biglycan is also a PG of the SLRP family with two DS/CS chains linked to serine residues in the protein core, homologous to the decorin protein (Fisher et al., 1989). Biglycan binds various growth factors to modulate cell signaling pathways, and disruption of the biglycan gene results in osteopenia with decreased growth and bone mass (Young et al., 2002). Biglycan is expressed throughout the corneal stroma (Figure 4) with high expression levels during early developmental stages, which decrease to very low levels in the mature cornea (Zhang et al., 2009). However, normal collagen fibrils and stromal structures are found in biglycan-*null* mice (Zhang et al., 2009). Since decorin and biglycan interact with a similar region on collagen when regulating lateral fibril growth, studies in decorin and biglycan double knockout mice have shown that decorin and biglycan have a synergistic effect producing abnormal fibril morphology resulting in additive deficiency in skin and bone (Young et al., 2002; Bi et al., 2005). Thus, there is a compensatory effect between decorin and biglycan with regard to regulating collagen fibril organization. Decorin and biglycan double knockout mice also show large irregular collagen fibrils throughout the corneal stroma, especially in posterior regions (Zhang et al., 2009), confirming compensatory expression in single decorin- or biglycan-*null* mice. Accordingly, in decorin-*null* mice there is higher than normal expression of biglycan in the cornea, although in biglycan-*null* corneas there is not a significant change in decorin expression (Zhang et al., 2009). This suggests that decorin has a primary role in corneal fibrillogenesis with some contribution from biglycan, which can compensate for the loss of decorin (Zhang et al., 2009).

## Heparan Sulfate Proteoglycans (HSPGs)

Heparan sulfate and HEP are synthesized as a polymer made up of repeating disaccharide units composed of GlcA and GlcNAc polymerized by glycosyltransferase enzymes called exostosins (Exts). HS/HEP are sequentially modified during and shortly after biosynthesis. The NDST enzyme creates different degrees of sulfation by *N*-deacetylation and *N*-sulfation of GlcNAc, which creates domains of unmodified *N*-acetylated disaccharides (NA domains), *N*-sulfated disaccharide units (NS domains), and alternating *N*-acetylated and *N*-sulfated disaccharides (NS/NA domains) (Esko and Lindahl, 2001). These modifications create binding sites for different proteins and also enable further enzymatic modification of the HS chain, such as C5-epimerization of GlcA to IdoA and *O*-sulfation of both residues (Esko and Lindahl, 2001; Ledin et al., 2004). Abnormalities in the biosynthesis of HS chains can lead to developmental defects ranging from embryonic lethality to tumor formation and altered organ-system development (Poulain and Yost, 2015).

In order to study the role of HS/HEP and HS/HEP sulfation *in vivo* and also understand how the various enzymes involved in HS biosynthesis and modifications are regulated, *null* and floxed mice have been generated for many of the biosynthetic enzymes. Many of these mice have been used in eye related studies. HS was ablated from corneal epithelial cells using a cytokeratin 14-rtTA (K14RtTA diver mouse);TetO-cre to remove exostosin 1 gene (*Ext1*), which resulted in defective corneal stratification and delayed wound healing due to loss of tight junctions (Coulson-Thomas et al., 2015). A similar phenotype was also observed in extracellular endosulfatase Sulf1-*null* mice (Maltseva et al., 2013). Studies have shown that the number and sulfation pattern of HS on HSPGs, such as syndecans, GPCs, perlecan and agrin, can modify their function (Mertens et al., 1996; Langford et al., 1998; Poulain, 2015).

Syndecans are a family of type-I transmembrane proteins that consists of syndecan-1, syndecan-2, syndecan-3 and syndecan-4. They are cell surface PGs that can carry three to five HS chains or CS chains and are involved in cell proliferation, migration and cell-matrix interactions (Bernfield et al., 1999). Syndecan-1 and syndecan-3 have GAG binding sites at both ends of the ectodomain, whereas syndecan-2 and syndecan-4 have them at the distal region only (Elenius and Jalkanen, 1994). Syndecan-1 is mostly expressed in the epithelium and syndecan-2 (fibroglycan) is expressed in vascular endothelium mediating angiogenic sprouting during development. Syndecan-3 (*N*-syndecan) is expressed in Schwann cells and on axons in neurons migrating along nerve bundles (Toba et al., 2002; Hienola et al., 2006). Syndecan-4 is expressed ubiquitously and plays a role in fibroblast migration, angiogenesis and wound healing (Morgan et al., 2007). In the cornea, syndecan-1 has been shown to play a role in corneal epithelial homeostasis, wound healing, and more recently, reinnervation (Pal-Ghosh et al., 2017). Syndecan-1-*null* mice with a BALB/c background are viable and fertile, with transparent corneas but altered intraepithelial corneal nerve morphology (Stepp et al., 2002). Loss of syndecan-1 causes delayed wound healing in skin and cornea due to defective migration and integrin expression in epithelial cells (Stepp et al., 2002). Altered integrin activity and TGFβ1 signaling makes epithelial cells isolated from syndecan-1-*null* mice more adhesive than wild-type cells (Stepp et al., 2010). The syndecan-1-*null* corneal epithelial cells assemble an abnormal ECM which can affect their interaction with intraepithelial nerves, resulting in altered morphology and regeneration after injury (Pal-Ghosh et al., 2017). Syndecan-1 deficient mice also present increased corneal neovascularization and adhesion of leukocytes to endothelial cells after alkali burn injury, indicating that syndecan-1 negatively regulates leukocyte-mediated angiogenesis (Götte et al., 2002). Interestingly, the overexpression of syndecan-1 in transgenic mice also delays wound closure and re-epithelialization due to shedding of soluble syndecan-1 ectodomains and inhibition of cell proliferation at wound edges (Elenius et al., 2004). Syndecan-4 is ubiquitous and necessary for focal adhesion formation by the interaction of fibronectin, integrin and intracellular components (Echtermeyer et al., 2001). After skin injury, syndecan-4 is upregulated in fibroblasts and endothelial cells within granulation tissue, and syndecan-4-deficient mice present delayed wound healing and reduced vessel size within granulation tissue of the wound bed (Echtermeyer et al., 2001), indicating that syndecan-4 regulates fibroblast migration, wound contraction and angiogenesis. However, a similar role for syndecan-4 in corneal wound healing has not been demonstrated.

Glypicans are a family of HS PGs linked to the cell surface by GPI-anchors. Studies have shown that GPCs influence growth factor activity in signaling pathways associated with developmental morphogenesis and disease processes in *Drosophila*, zebrafish, *Xenopus*, and mammals. The location of the HS chain insertion site in the C-terminus is conserved in all GPCs (Veugelers et al., 1999). In *Drosophila*, the GPC genes are called *division abnormally delayed* (*dally*) and *dally-like protein* (*dlp*). Mutation studies in the *dally* gene indicate that it is necessary for G2-M progression in the cell cycle during eye development (Nakato et al., 1995; Jackson et al., 1997). Ten GPC genes have been identified in zebrafish (Gupta and Brand, 2013) and six (GPC1-6) in mammals, which have all been shown to be important in embryonic development (Brown and Waneck, 1992). The expression and function of GPCs in the ocular surface remains to be established, and further research in this area is needed.

Perlecan is a major basement membrane-specific HS PG 2 (HSPG2) with four potential sites for HS/CS side chain attachment (Iozzo, 2005). Constitutive loss of perlecan is embryonic lethal due to heart and brain defects with a few animals developing chondrodysplasia after birth (Arikawa-Hirasawa et al., 1999; Costell et al., 1999). In order to study the role of perlecan in adult mice, researchers have used mutant mice lacking exon 3 of the perlecan gene, which have smaller eyes (microphthalmia) and develop lens degeneration (congenital cataract) (Rossi et al., 2003). In the human cornea, perlecan was detected by immunofluorescence in the entire corneal and conjunctival epithelial basement membrane (Figure 4), and also between the endothelium and Descemet’s membrane (Ljubimov et al., 1995; Torricelli et al., 2015). *Hspg2^–/–^*-Tg are perinatal lethality rescued mice produced in a perlecan-*null* (Hspg2^–/–^) genetic background that express recombinant perlecan specifically in the cartilage and not in other tissues (Ishijima et al., 2012). *Hspg2^–/–^*-Tg mice have small eyes with thinner corneal epithelial wing cell layers and significantly reduced expression of corneal epithelial differentiation markers (Inomata et al., 2012). Similarly, thin and poorly organized epidermis was formed in engineered human skin when perlecan expression was disrupted in keratinocytes (Sher et al., 2006). The HS side chains of perlecan have also been suggested to play a role in corneal epithelial homeostasis and wound healing since transgenic mice lacking the *Ext1* gene in epithelial cells have an increased rate of epithelial cells sloughing off due to disrupted tight junctions and a reduced number of cell-basement membrane adhesion complexes (Coulson-Thomas et al., 2015). If the basement membrane of the mouse cornea is exposed, perlecan can act as a binding site for *Pseudomonas aeruginosa*; however, when the HS side chains are removed by heparanase, bacterial binding decreases (Chen and Hazlett, 2000). The regeneration of normal basement membrane relies on the production of perlecan during corneal wound healing, which is modulated by IL-1 and TGF-β (Saikia et al., 2018). Perlecan has also been shown to be expressed by stromal keratocytes and is upregulated after epithelial injury (Torricelli et al., 2015).

Agrin is a multidomain HSPG expressed as an alternatively spliced protein, with the different isoforms playing important roles during embryonic development, especially of the nervous system (Stetefeld et al., 2004). In zebrafish, agrin morphants that were generated using antisense morpholino oligonucleotides show that agrin is important for neurite outgrowth during retina development (Kim et al., 2007; Liu et al., 2008). Since the disruption of this neuromuscular development process causes perinatal mortality in agrin-deficient mice, the role of agrin has been studied in transgenic mice by tissue-specific inactivation of agrin (Gautam et al., 1996) and overexpression of agrin (Fuerst et al., 2007). Alteration of agrin expression presents highly variable developmental defects in the eye including anophthalmia, microphthalmia, optic nerve hypoplasia, optic stalk coloboma, vitreous vessel persistence, and adhesion of iris and lens to the cornea (Fuerst et al., 2007). In the ocular surface, agrin is highly expressed in the limbal regions partially colocalized with limbal stem cells (ABCG2 and p63-positive cells), and the expression of agrin decreases toward the conjunctiva (weakly expressed) and central cornea (absent or weakly expressed) (Figure 4) (Schlötzer-Schrehardt et al., 2007). A recent report has shown that agrin promotes limbal stem cell proliferation and corneal wound healing via the Hippo-Yap signaling pathway (Hou et al., 2020). Conditional knockout of agrin in the cornea could potentially help further our understanding of the role of agrin in supporting limbal stem cells.

Collagen XVIII is a basement membrane collagen/PG with HS side chains that is abundant in the eye, skin, muscles, kidney, lungs and blood vessels (Halfter et al., 1998). In the posterior segment of the eye, collagen XVIII immunolabeling is present in the retinal inner limiting membrane, retinal pigmented epithelium and Bruch’s membrane (Halfter et al., 1998; Zatterstrom et al., 2000; Fukai et al., 2002). Collagen XVIII anchors collagen fibrils to the inner limiting membrane, and the inactivation of collagen XVIII in mice causes vitreous separation from the retina. In humans, a collagen XVIII loss-of-function mutation causes Knobloch syndrome, characterized by high myopia, vitreoretinal degeneration and retinal detachment in humans (Fukai et al., 2002). When cleaved, the C-terminal fragment of collagen XVIII generates endostatin, an angiogenesis inhibitor (O’Reilly et al., 1997). Mice lacking collagen XVIII and endostatin show abnormal retinal vessel outgrowth and delayed hyaloid vessel regression after birth (Zatterstrom et al., 2000; Lin et al., 2001). These findings indicate that collagen XVIII can be targeted for the regulation of angiogenesis in the eye. In the anterior segment of the eye, collagen XVIII has been immunolocalized in the corneal epithelium, conjunctival epithelial basement membrane, corneal nerve basement membrane, Descemet’s membrane, limbal and conjunctival capillaries, ciliary epithelium and lens capsule (Zatterstrom et al., 2000; Fukai et al., 2002; Sakimoto et al., 2008). The expression of collagen XVIII and endostatin is altered in different types of corneal injuries and pathologies. The immunolabeling of collagen XVIII and endostatin is irregular in the epithelial basement membrane of keratoconic corneas and highly increased in the stroma of scarred corneas (Määttä et al., 2006). Similarly, collagen XVIII expression increases in the stroma and epithelial basement membrane after linear corneal incision (Kato et al., 2003). Following excimer laser keratectomy, collagen XVIII immunoreactivity is absent in the epithelial basement membrane in wild-type mice (Kato et al., 2003), and corneal reinnervation is impaired in collagen XVIII knockout mice (*col18a1^–/–^*) with decreased corneal neurite extension (Sakimoto et al., 2008).

## Heparin

Heparin is a heavily sulfated GAG synthesized upon a serglycin core protein found only in mast cells and some hematopoietic cells (Kolset and Gallagher, 1990). It has the highest negative charge density among all biological macromolecules due to the fact that it contains trisulfated disaccharides (Figure 1) (Capila and Linhardt, 2002). During biosynthesis, heparin is released from serglycin by tissue proteases followed by endoglucuronidase (Kolset and Gallagher, 1990; Capila and Linhardt, 2002). Although HS and HEP have the same core structure and most HS-binding proteins can also bind to heparin, they have different levels of sulfation and epimerization (Kolset and Gallagher, 1990). Heparin is widely used for its anticoagulant effects but recent experimental and clinical evidence shows that it also has anti-viral, anti-inflammatory and wound healing properties (Galvan, 1996; Olczyk et al., 2015; He et al., 2019; Çevirme et al., 2020; Gavioli et al., 2020; Magro, 2020; Zheng et al., 2020). Heparin exhibits anti-inflammatory activities and has an effect on the hemostatic phase of wound healing by various mechanisms such as inhibition of leukocyte recruitment, reduced vascular permeability, neutralization of cationic mediators, and inhibition of heparanase (Olczyk et al., 2015). The wound healing effect of heparin is mostly through binding and regulating the activity of enzymes and growth factors such as heparin-binding EGF-like growth factor (HB-EGF), which is known to promote re-epithelialization of murine partial-thickness burns, increase keratinocyte proliferation and heal intestinal anastomotic wounds (Cribbs et al., 1998; Radulescu et al., 2011). Topical application of heparin in rabbit tracheal autografts has also shown effective tissue repair after surgery (Sen et al., 2009). In eyes, polymer pellets impregnated with heparin and cortisone inhibit corneal vascularization in rabbit models of corneal injury and transplantation (Nikolic et al., 1986). Heparin eye drops have also been shown to reduce the occurrence of conjunctival pseudomembrane in the ocular surface after a chemical injury with paraquat (Jian-Wei et al., 2017).

## Hyaluronan

Hyaluronan is composed of alternating residues of β-D-(1 → 3)GlcA and β-D-(1 → 4)-GlcNAc and is neither sulfated nor covalently attached to a protein core. It is synthesized by HAS enzymes, namely HAS1, HAS2, and HAS3, and is directly extruded into the ECM without epimerization or sulfation (Itano et al., 1999; Hascall and Esko, 2015). It has a very high negative charge that can attract sodium and retain water. HA is ubiquitous and serves a variety of functions in vertebrate tissues such as providing cushioning and lubrication in synovial joints (Balazs et al., 1967), forming hydrated complexes in skin and cartilage (Hardingham and Fosang, 1992), binding proteins and growth factors involved in cell signaling important in regulating homeostasis, tissue repair, inflammation, and cancer (Fraser et al., 1997; Schwertfeger et al., 2015).

Studies have used biotinylated-HA binding protein to detect HA in various tissues of the eye. In the posterior segment of the eye, HA has been detected in the vitreous humor, neurosensory retina, retinal pigmented epithelium, Bruch’s membrane and choroid (Clark et al., 2011). In the anterior segment of normal eyes, HA has been detected in the aqueous humor, trabecular meshwork, iris stroma, lens capsule, corneal endothelium, conjunctival epithelium and limbal epithelium (Figure 4) (Härfstrand et al., 1992; Gong et al., 1994; Lerner et al., 1998; Gartaganis et al., 2001; Gesteira et al., 2017). Although studies have shown that the biological function of HA is dictated by its molecular weight, the molecular size of HA in these tissues have not been determined.

The spatial and temporal expression pattern of HA has been shown to play important roles in development, homeostasis, and diseases of the ocular surface. During development, HA is present throughout the cornea and facilitates the movement of mesenchymal cells and lymphatic vessel growth into the cornea (Toole and Trelstad, 1971; Hart, 1978; Sun et al., 2019). HA is cleared from the central cornea by hyaluronidase enzyme digestion during later stages of development accompanied by regression of lymphatic vessels (Toole and Trelstad, 1971; Sun et al., 2019). To date, HA is the only GAG that has been identified to be decreased in aged human corneas when compared to corneas from younger individuals (Pacella et al., 2015). In adult eyes, HA is highly expressed in the limbal epithelium, especially in the basal layer (Gesteira et al., 2017). HA is also expressed in the conjunctival epithelium and very low expression levels are detected in the corneal epithelium of murine (Gesteira et al., 2017; Sun et al., 2019), bovine (Gong et al., 1994), and human (Lerner et al., 1998) corneas. This HA specific matrix in the limbus is important for the limbal stem cell niche and the disruption of this HA matrix leads to altered LSC phenotype (Gesteira et al., 2017). HA is significantly increased in the cornea after corneal injuries like penetrating wounds, excimer laser surgeries and alkali burns (Hassell et al., 1983; Fagerholm et al., 1992; Fitzsimmons et al., 1997; Gesteira et al., 2017; Sun et al., 2019). This increase in HA is believed to be necessary for binding provisional fibronectin and stimulating corneal epithelial migration for corneal wound healing (Nakamura et al., 1992; Camillieri et al., 2004). HA expression in the cornea after alkali burn also regulates corneal lymphangiogenesis by acting as a substrate for lymphatic vessel growth (Sun et al., 2019). The precise mechanism of action of HA and involvement in cell signaling pathways in the ocular surface remain elusive. Although HA is currently widely used in ophthalmic products, the actual mechanism of action and long-term effects of HA in the eye are not clearly understood.

## Involvement of Proteoglycans and Glycosaminoglycans in Corneal Pathology

Glycosaminoglycans and PGs have been associated with numerous diseases of the ocular surface. Changes in the levels of GAGs and PGs can be the primary cause of a disease, such as in mucopolysaccharidosis (MPS), cornea plana and macular corneal dystrophy, or secondary to the disease, such as in corneal scarring and keratoconus. MPS is a family of lysosomal disorders caused by a mutation in a gene encoding enzymes involved in the sequential degradation of GAGs, which leads to their gradual build-up in cells and tissues (Cantz and Gehler, 1976; Freeze et al., 2015). MPS patients suffer from numerous ocular manifestations, such as corneal clouding, retinal dystrophy and retinal degeneration (Javed et al., 2017). In more severe cases flat and thickened corneas have been noted using anterior segment optical coherence tomography (Matoba et al., 2020). The current treatment modalities for MPS include enzyme replacement therapy and bone marrow transplantation, which significantly delay progression of the disease but are not effective in preventing corneal clouding (Vogler et al., 1996; Rohrbach and Clarke, 2007; Clarke, 2008; Papadia et al., 2011; Beck, 2018). Ultimately, patients with severe corneal clouding require corneal transplantation in order to regain vision, thus, there is an unmet need to develop treatment options to prevent the build-up of GAG products within the cornea. Importantly, research is also warranted to explore ocular manifestations early in the disease since ophthalmologists and optometrists are often the first to suspect a MPS diagnosis (Del Longo et al., 2018). In the cornea, the accumulation of GAGs is primarily evident in keratocytes, which over time causes them to swell and lose their fibroblast-like shape (Young et al., 2007; Quantock and Young, 2008; Coulson-Thomas et al., 2014). Granules, containing primarily partially degraded GAGs, are gradually deposited throughout all corneal layers disrupting the orthogonal arrangement of the collagen fibrils as the disease progresses (Fahnehjelm et al., 2012). The use of gene therapy for treating MPS has been tested in the corneas of human cadavers, mice, rabbits and dogs (Thomas et al., 2012; Vance et al., 2016; Miyadera et al., 2020). Strikingly, AAV-opt-IDUA administered via a single intrastromal injection in a spontaneous MPS I canine model significantly cleared the accumulated GAGs within the corneal tissue and prevented further accumulation (Miyadera et al., 2020). Cell therapy has also been explored for treating the corneal defects associated with MPS. Umbilical cord mesenchymal stem cells have been shown to significantly decrease the accumulation of GAGs throughout the corneal tissue and within keratocytes (Coulson-Thomas et al., 2013).

Macular corneal dystrophy is a rare condition characterized by the build-up of poorly sulfated or non-sulfated KS chains (poly-lactosamine chains) within keratocytes and the corneal endothelium (Funderburgh et al., 1990; Lewis et al., 2000). Clinical manifestations can resemble certain MPS subtypes, and include corneal clouding (Funderburgh et al., 1990; Lewis et al., 2000). Macular corneal dystrophy has mostly been correlated with mutations in the carbohydrate sulfotransferase 6 (CHST6) gene, which encodes the enzyme carbohydrate sulfotransferase 6, which catalyzes the transfer of a sulfate group to the GlcNAc residue of KS (Hasegawa et al., 2000). To date, over 130 mutations have been reported in the CHST6 gene and linked to macular corneal dystrophy (Hasegawa et al., 2000; El-Ashry et al., 2002; Sultana et al., 2005; Patel et al., 2011; Nejat et al., 2018).

Cornea plana is a rare disorder that is characterized by small flat corneas, hypermetropia, haze in the corneal limbal region and corneal clouding (Ebenezer et al., 2005; Khan et al., 2006a,b). To date 15 different mutations in the keratocan (KERA) gene have been reported in families with cornea plana (Khan et al., 2004, 2005, 2006a,b; Liskova et al., 2007; Huang et al., 2019). In fact, mutations in the KERA gene have been associated with the more severe forms of cornea plana (Pellegata et al., 2000). Interestingly, keratocan knock-out mice present narrow anterior chamber angles and thin corneal stroma, clinical features that resemble cornea plana (Huang et al., 2019). Changes in GAG and PG levels, and also changes in GAG processing enzymes, have also been reported in other ocular pathologies, such as reduced levels of sulfated KS in pellucid marginal degeneration and ocular autoimmune diseases (Funderburgh et al., 1990; Woodward et al., 2019).

As mentioned, changes in the expression of GAGs and PGs can also occur secondary to many diseases. These changes can often play a role in the pathogenesis of the disease and, therefore, can often serve as a pharmaceutical target. Keratoconus is a common progressive corneal ectasia that drastically impairs vision due to the bulging, thinning and scarring of the cornea (Rabinowitz, 1998). Although the etiology of keratoconus is poorly understood and research indicates it is a multifactorial degenerative disease, changes to the ECM are believed to play a key role in its pathogenesis. There are limited treatments available for keratoconus, primarily due to the fact that so little is known about the disease itself; however, collagen cross-linking has emerged as a ground breaking treatment option to slow the progression of corneal distortions (Kobashi and Rong, 2017). Studies have indicated that the expression of GAGs and PGs, such as sulfated KS, decorin, lumican, and fibromodulin, are down-regulated in keratoconus corneas compared to healthy controls (Funderburgh et al., 1990; García et al., 2016; Foster et al., 2018). Further research is needed to understand the role GAGs and PGs play in keratoconus and also to understand their involvement in collagen cross-linking therapy. Importantly, riboflavin and ultraviolet-A collagen cross-linking has been shown to cross-link certain PG core proteins culminating in the formation of higher molecular weight macromolecules (Zhang et al., 2011). Specifically, mimecan and decorin form cross-links with collagen both *in vitro* and *ex vivo*, and keratocan and lumican core proteins strongly inhibit collagen cross-linking *in vitro*, while glycosylated keratocan and lumican form cross-links with collagen *ex vivo* (Zhang et al., 2011).

Another disease that has been shown to cause secondary changes to GAG and PG expression is Marfan syndrome. Marfan syndrome is a disease that affects the ECM of connective tissues due to mutations in the gene that encodes fibrillin-1, necessary for the formation of elastic fibers (Feneck et al., 2020). Patients suffer many ocular manifestations, including thinned and flattened corneas. Decorin expression has been shown to be significantly decreased in the corneas of a mouse model of Marfan syndrome and fibroblasts from a patient with Marfan syndrome (Superti-Furga et al., 1992; Feneck et al., 2020). The Meek group speculate the decrease in decorin expression could be a consequence of the increased levels of TGF-β expression found in the corneas of individuals with Marfan disease (Feneck et al., 2020). However, whether the loss of decorin could further contribute to the pathogenesis of Marfan syndrome remains to be established.

Pterygium is a benign fibrovascular hyperplasia of the conjunctiva that gradually grows into the cornea. Although the cause of pterygium remains unknown, studies have suggested UV exposure as a major cause. KS (stroma), HS (epithelial layer of blood vessels) and DS (stroma) have been shown to be increased in pterygium tissue when compared to normal conjunctiva and have been speculated to potentially be involved in the hyperplastic process of pterygium (Georgakopoulos et al., 2019).

Dry eye is a common condition that is caused by an insufficient and/or unstable tear film that leads to an itchy feeling in the eye, foreign object sensation, stinging and/or burning sensation, red eyes, sensitivity to light, blurry vision, fatigue, and, ultimately, inflammation, corneal erosions and compromised vision (Craig et al., 2017). Dry eye can be caused by reduced or altered secretion from lacrimal glands, which produce the aqueous layer of the tear film, Meibomian glands, which produce the lipid layer of the tear film, or conjunctival cells, which produce the mucin layer (Bron et al., 2017). Unfortunately, the etiology of dry eye remains unknown and treatment options are limited to palliative care (Bron et al., 2017; Jones et al., 2017). Given the basic characteristics of GAGs, which are highly hydrophilic, and have high viscosity and low compressibility, they have emerged as powerful lubricating agents in artificial tears. The anti-inflammatory characteristics that have been attributed to GAGs and PGs, such as high molecular weight HA (HMWHA), makes them even more attractive candidates for use in treating dry eye. The number of studies using HA, chemically modified HA and HA analogs has increased exponentially over the past few years (Salwowska et al., 2016; Ang et al., 2017; Pinto-Fraga et al., 2017). Lubricin is also emerging as a powerful agent in treating dry eye (Lambiase et al., 2017; Regmi et al., 2017). Despite the countless studies dedicated toward establishing the therapeutic benefits of using HA and lubricin for treating ocular conditions, such as dry eye, and also the fact that eye drops containing HA are widely sold around the world, limited studies have investigated the long-term effects of continuous exposure of the ocular surface to HA and lubricin. We have recently shown that alterations in HA expression in keratin 14 positive cells leads to Meibomian gland and conjunctival hyperplasia (Sun et al., 2018). Also, increased expression of HA throughout the cornea leads to lymphangiogenesis (Sun et al., 2019). This work supports the need for more research focused on understanding the long-term effects of the topical administration of GAGs/PGs to the ocular surface. Some interesting studies have suggested that changes in HS and HA expression can lead to changes within lacrimal glands and Meibomian glands, and the expression of HS glycosyltransferases, such as HS2ST, HS3ST, and EXTL2, have been shown to be significantly downregulated in dry eye (Pan et al., 2008; Mantelli et al., 2009; Qu et al., 2011; Patel et al., 2017; Sun et al., 2018).

For many years the cornea has been a valuable tool for studying the processes of wound healing and scarring due to the fact it is transparent, easily accessible and has an anatomy that is highly conserved inter- and intraspecies. Corneal injury leads to a cascade of events that are aimed at rapidly repairing the damaged tissue and preventing infection. Although a similar cascade of events is triggered upon corneal injury, the wound healing response varies significantly based on the insult, specifically, damage and/or perforation of the basement membrane, damage to the stroma, delayed epithelial wound closure and infection can all lead to an exacerbated inflammatory response (Ishizaki et al., 1993; Wilson et al., 2001; Torricelli and Wilson, 2014). Upon injury, corneal epithelial cells migrate toward the injured area to rapidly resurface the wound, there is increased proliferation of peripheral epithelial cells and limbal stem cells to enable wound closure and stratification, and a subset of keratocytes differentiate into myofibroblasts (Wilson, 2020). These myofibroblasts play a pivotal role in corneal wound healing and scarring since they deposit a significant amount of ECM components that form a “provisional matrix,” which is needed during the wound healing process (Xue and Jackson, 2015). Excessive deposition of ECM components and/or failure to replace this “provisional matrix” and regenerate the stromal matrix leads to fibrosis/scarring and loss of vision (Jester et al., 1999; Hassell and Birk, 2010). Essentially, corneal scarring is caused by excessive disorganized stromal matrix remodeling that occurs after injury. These fibrotic changes have been shown to primarily take place following damage to the corneal epithelial basement membrane (Torricelli et al., 2016). Epithelium-derived growth factors such as TGF-β and platelet-derived growth factor (PDGF) along with inflammatory cytokines like interleukin-1 released from epithelial cells trigger apoptosis or transdifferentiation of keratocytes to myofibroblasts (Wilson et al., 2001; Torricelli et al., 2016). These myofibroblasts express CS/DS with longer chains and increased sulfation and KS with shorter chains and decreased sulfation when compared to normal keratocytes (Funderburgh et al., 2003). The change in structure of these GAG chains leads to modification of the PG profile in the stroma during stromal scar formation (Funderburgh et al., 2003; Saikia et al., 2018). During corneal epithelial wound healing, the loss of HA was shown to delay wound healing (Gesteira et al., 2017), while an increase in HA leads to corneal lymphangiogenesis and corneal scarring (Sun et al., 2019). The intensity of the inflammatory response and transdifferentiation of keratocytes into myofibroblasts, which together up-regulate the expression GAGs and PGs during the wound healing process, ultimately determine whether the corneal injury will result in restoration of transparency or fibrosis/scarring. Further studies are warranted to elucidate the role of these ECM components in corneal regeneration and to determine methods of intervention that could limit excessive ECM deposition and scarring.

## Summary

**FIGURE 7:**
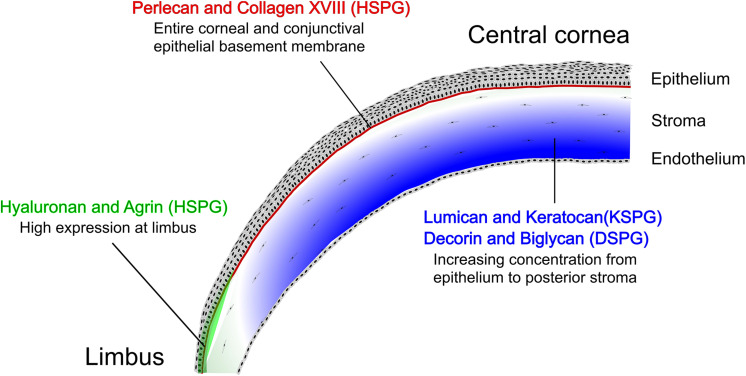
Representation of the major GAGs and PGs in the cornea and limbal region. Diagram of a cross-sectional view of the cornea and limbal region showing the distribution of major GAGs and PGs in the cornea. The different layers of the cornea and limbus are represented, specifically the epithelium (stratified squamous epithelium), stroma (ECM with sparse keratocytes) and endothelium. The basement membrane can be seen as a line (represented in green in the limbal region and red in the cornea) between the epithelium and the stroma.

Significant progress has been made in understanding the distribution and function of different types of GAGs and PGs in the development, homeostasis and pathology of the ocular surface. A schematic of the cornea and limbal region with the distribution of the major corneal PGs and GAGs is shown in Figure 7. A summary of the studies characterizing the expression profiles of the different GAGs and PGs has been provided in Table 1. Even though significant progress has been made in characterizing the distribution of GAGs and PGs throughout the ocular surface, the potential therapeutic uses of these molecules in ocular health and diseases are still largely unexplored and/or poorly understood.

**TABLE 1:** Summary of studies characterizing the expression profiles of the different GAGs and PGs in the cornea.

GAGs	Gene	Expression in ocular surface	Function/deficiency	References
Keratan sulfate	Lumican	Primarily expressed in the stroma. Expressed by stromal keratocytes and corneal epithelial cells (during wound healing).	Deficiency causes corneal opacity and thinning due to abnormal collagen fibril structure, and delayed corneal wound healing.	Chakravarti et al., 1998, 2000; Saika et al., 2000
	Keratocan	Expressed throughout the corneal stroma by keratocytes only.	Deficiency causes corneal thinning and abnormal collagen fibril spacing. Mutation causes cornea plana congenital.	Pellegata et al., 2000; Kao and Liu, 2002; Carlson et al., 2005
	Fibromodulin	Expressed in the stroma of the peripheral cornea, limbus and sclera.	Deficiency causes a change in corneal curvature and the axial length of the eye.	Chen et al., 2010
	Mimecan	Expressed in the corneal stroma.	Deficiency causes an increase in collagen fiber diameter in the cornea.	Beecher et al., 2005
Heparan sulfate	Syndecan-1	Expressed in the corneal epithelium.	Deficiency causes delayed wound healing and abnormal intraepithelial nerve terminals in the cornea, as well as increased corneal neovascularization after alkali burn. Overexpression inhibits cell proliferation at the wound edge.	Götte et al., 2002; Stepp et al., 2002, 2010; Elenius et al., 2004; Pal-Ghosh et al., 2017
	Perlecan	Expressed in the entire corneal and conjunctival epithelial basement membrane and also between the endothelium and Descemet’s membrane. Expressed by corneal epithelial cells and keratocytes.	Deficiency causes a thinner corneal epithelium with decreased expression of epithelial differentiation markers.	Ljubimov et al., 1995; Inomata et al., 2012; Torricelli et al., 2015
	Agrin	High expression at the limbus decreasing toward the central cornea and conjunctiva.	Deficiency can cause anophthalmia, microphthalmia, or iris adhesion to the cornea.	Fuerst et al., 2007; Schlötzer-Schrehardt et al., 2007
	Collagen XVIII	Expressed in the corneal epithelium, corneal and conjunctival epithelial basement membrane, corneal nerve, Descemet’s membrane, and limbal and conjunctival capillaries. Increased expression in the stroma after injury.	Deficiency causes impaired corneal reinnervation after injury.	Zatterstrom et al., 2000; Fukai et al., 2002; Kato et al., 2003; Määttä et al., 2006; Sakimoto et al., 2008
	Exostosin glycosyltransferase 1 (*Ext1*) enzymes	Primarily expressed in the corneal epithelium.	Deletion of *Ext1* in the corneal epithelium causes defective corneal stratification and delayed wound healing due to loss of tight junctions.	Coulson-Thomas et al., 2015
Chondroitin sulfate	Lubricin	Expressed in corneal and conjunctival epithelia.	Functions as a boundary lubricant. Deficiency causes the dry eye phenotype.	Cheriyan et al., 2011; Schmidt et al., 2013
	Aggrecan	Expressed in corneal and conjunctival epithelia, low expression in the anterior corneal stroma and moderate expression in the scleral stroma.	Increased expression in the stroma of sclerocornea.	Bouhenni et al., 2013
	Versican	Expressed in the subepithelial region of the anterior limbus.		Schlötzer-Schrehardt et al., 2007
Dermatan sulfate	Decorin	Expressed throughout the corneal stroma.	Plays a role in corneal fibrillogenesis. Mutation causes congenital stromal dystrophy of the cornea.	Danielson et al., 1997; Bredrup et al., 2005; Rødahl et al., 2006; Zhang et al., 2009
	Biglycan	Expressed throughout the corneal stroma.	Plays a role in corneal fibrillogenesis.	Zhang et al., 2009
Hyaluronan	Hyaluronan synthases 1, 2, and 3	Highly expressed in the limbus and to some point in the conjunctiva.	Regulates limbal stem cell differentiation. Facilitates corneal epithelial wound healing. Regulates corneal lymphangiogenesis.	Gong et al., 1994; Lerner et al., 1998; Gesteira et al., 2017; Sun et al., 2019

## Author Contributions

SP and VC-T wrote the manuscript. SP, TG, and VC-T made the figures. TG and YC-T contributed toward parts of the manuscript and read and approved the final version. All authors contributed to the article and approved the submitted version.

## Conflict of Interest

TG is the co-founder and was employed by Optimvia, LLC. The remaining authors declare that the research was conducted in the absence of any commercial or financial relationships that could be construed as a potential conflict of interest.

## Abbreviations

ALK5: activin linked kinase 5
Asn: asparagines
Cmd: cartilage matrix deficiency
CS: chondroitin sulfate
CSPGs: chondroitin sulfate proteoglycans
DS: dermatan sulfate
DSPGs: dermatan sulfate proteoglycans
ECM: extracellular matrix
ERK: extracellular signal-regulated kinase
Ext1: exostosin glycosyltransferase 1
FAK: focal adhesion kinase
GAGs: glycosaminoglycans
Gal: galactose
GalNAc: *N*-acetylgalactosamine
GlcA: glucuronic acid
GlcNAc: *N*-acetylglucosamine
GlcNAc6ST: *N*-acetylglucosamine -6-*O*-sulfotransferase
GPCs: glypicans
GPI: glycosylphosphatidylinositol
HA: hyaluronan
HAS: hyaluronan synthase
HEP: heparin
HS: heparan sulfate
HSPGs: heparan sulfate proteoglycans
IdoA: iduronic acid
JNK: c-JunN-terminal kinase
KS: keratan sulfate
KSPGs: keratan sulfate proteoglycans
LPS: lipopolysaccharides
MMP: matrix metalloproteinase
NDST: glucosaminyl *N*-deacetylase/*N*-sulfotransferase
PAPS: 3 ′ -phosphoadenosine-5 ′ -phosphosulfate
PGs: proteoglycans
PRG4: proteoglycan 4
Ser: serine
SLRPs: small leucine-rich proteoglycans
TGF- β: transforming growth factor beta
TGFBIP: TGF beta-induced protein
Thr: threonine
TLR4: toll-like receptor 4.
